# *parkin *mutation dosage and the phenomenon of anticipation: a molecular genetic study of familial parkinsonism

**DOI:** 10.1186/1471-2377-5-4

**Published:** 2005-02-22

**Authors:** Parvoneh Poorkaj, Lina Moses, Jennifer S Montimurro, John G Nutt, Gerard D Schellenberg, Haydeh Payami

**Affiliations:** 1 Department of Psychiatry and Behavioral Sciences, University of Washington, Seattle, USA; 2 Genomics Institute, Wadsworth Center, New York State Department of Health, Albany, NY, USA; 3 Department of Neurology, Oregon Health & Science University, Portland, OR, USA; 4 Departments of Neurology and Pharmacology, University of Washington, and Geriatric Research Education Clinical Center, Veterans Affairs Puget Sound Health Care System, Seattle, WA, USA

## Abstract

**Background:**

*parkin *mutations are a common cause of parkinsonism. Possessing two *parkin *mutations leads to early-onset parkinsonism, while having one mutation may predispose to late-onset disease. This dosage pattern suggests that some *parkin *families should exhibit intergenerational variation in age at onset resembling anticipation. A subset of familial PD exhibits anticipation, the cause of which is unknown. The aim of this study was to determine if anticipation was due to *parkin *mutation dosage.

**Methods:**

We studied 19 kindreds that had early-onset parkinsonism in the offspring generation, late-onset parkinsonism in the parent generation, and ≥ 20 years of anticipation. We also studied 28 early-onset parkinsonism cases without anticipation. Patients were diagnosed by neurologists at a movement disorder clinic. *parkin *analysis included sequencing and dosage analysis of all 12 exons.

**Results:**

Only one of 19 cases had compound *parkin *mutations, but contrary to our postulate, the affected relative with late-onset parkinsonism did not have a *parkin *mutation. In effect, none of the anticipation cases could be attributed to *parkin*. In contrast, 21% of early-onset parkinsonism patients without anticipation had *parkin *mutations.

**Conclusion:**

Anticipation is not linked to *parkin*, and may signify a distinct disease entity.

## Background

Mutations in the *parkin *gene are a common cause of parkinsonism. *parkin *was originally discovered as the cause of autosomal recessive juvenile parkinsonism [1]. However, recent reports suggest that not all *parkin *mutations are recessive [2-5], nor is age at onset always early [6-9]. Several studies have found heterozygous mutations in patients with late onset parkinsonism, suggesting that a single *parkin *mutation predisposes to later disease onset [8-10]. Collectively, these reports imply that *parkin *may exert a dosage effect in which possession of two mutations (homozygous or compound heterozygous) leads to early-onset parkinsonism, while possession of one normal and one mutant *parkin *(heterozygous) increases the risk for late-onset parkinsonism.

Several groups have reported what appears to be genetic anticipation in parkinsonism [11-13]. In the kindreds studied by them, the parent generation had typical late-onset parkinsonism and the individuals in the offspring generation developed the disease at much earlier ages. Barring the possibility that this pattern is an artifact of ascertainment bias (which cannot be ruled out until a biological mechanism is found), the intergenerational difference in onset age may be indicative of one of several mechanisms. The most common cause of anticipation is triplet repeat expansions. In a few parkinsonism families, disease has been shown to segregate with pathogenic expansions in SCA loci, but the search for other expansion loci in familial parkinsonism has been unsuccessful [14-16]. Other mechanisms that could result in anticipation include: change in mitochondrial heteroplasmy, which can affect disease severity and age at onset; a modifier gene that segregates independently of the disease gene; and parent/child exposure to a toxin. Gene dosage for dominant mutations can also mimic anticipation. For example, Familial Hypercholestrolemia is an autosomal dominant disorder in which heterozygotes develop the disease after the 4^th ^decade of life, whereas homozygotes show symptoms at much younger ages, sometimes at birth (OMIM *143890).

Is anticipation in parkinsonism related to *parkin *mutation dosage? We hypothesized that some *parkin *mutations are dominant: heterozygotes have incomplete penetrance and may develop late-onset parkinsonism, whereas homozygotes and compound heterozygotes have accelerated disease leading to early-onset parkinsonism. This unifying hypothesis was attractive because if true, it could explain the reported variations in mode of inheritance of *parkin *(recessive in some families, dominant in others), the range in age at onset from juvenile to late onset, and the significantly earlier onset in some of the children of affected parents. To test this hypothesis, we studied *parkin *in 19 kindreds with early-onset parkinsonism in the index generation, late-onset parkinsonism in the parent generation, and exhibited ≥ 20 years of anticipation.

## Methods

Families were recruited from a movement disorder clinic. The probands and affected family members had the clinical diagnosis of idiopathic Parkinson's disease (here referred to as parkinsonism due to lack of autopsy confirmation). Diagnosis was made by a neurologist according to the British Parkinson's Disease Brain Bank criteria except that family history was not an exclusion criterion [17]. These families were identified through an index case (a clinic patient) who reported a family history of parkinsonism. Positive family history was defined as patient reporting a first or second degree relative with parkinsonism, although we did inquire about more distant relatives as well. Clinic patients were enrolled sequentially and their affected relatives were subsequently identified and enrolled. For affected relatives we obtained medical records or personally examined them when possible. In some families the index case had early onset and the relative had late onset, in others, the index case had late onset and the relative with early onset was subsequently identified. The study was approved by the Institutional Review Boards at the participating institutions.

DNA was extracted from blood using standard protocol. Genotyping was blind to phenotype. To identify point mutations, we sequenced both DNA strands of all 12 exons. Exons and 50–100 bp of flanking intronic sequences were PCR-amplified [1], agarose gel-purified (Gene-clean III, Bio101), and directly sequenced by dye-terminator cycle sequencing (ABI, Big-Dye) using an ABI377 sequencer. To identify exon deletions and duplications, we analyzed gene dosage using real-time fluorescence-based PCR (ABI 7700 Sequence Detector). Amplification of subject genomic DNA was performed using fluorescently labeled probes (5' FAM or VIC, 3' TAMRA) and Taqman Universal PCR Mix (ABI) [4,18]. *parkin *exon amplifications were multiplexed under standard conditions with an 84-bp fragment of a single-copy human β-actin gene (Genbank accession number XM_004814) as an internal control. A standard curve was generated for each *parkin *exon and for β-actin using 0, 5, 15, 55 and 220 ng of control human genomic DNA. The number of PCR cycles required before the ABI 7700 detects each *parkin *exon product (CT value) was plotted against the corresponding exon standard curve, thus calculating the relative *parkin *copy number. The copy number for each exon was normalized to the single-copy actin gene within each multiplexed reaction and to a normal control reference individual, allowing an estimate of the number of copies of *parkin*. Optimal threshold levels for each primer set were maintained between plate analyses. All samples were analyzed in triplicate.

## Results

Nineteen kindreds were chosen for *parkin *analysis based on having parkinsonism in two consecutive generations, late-onset parkinsonism in the parent generation (onset ages 59 to 89 years, mean 71.0 ± 8.5), early-onset parkinsonism in the offspring generation (onset ages 8 to 47 years, mean 37.2 ± 9.8), and ≥ 20 years of anticipation as measured by the difference in mean ages at onset in two generations. In the younger generation, 2 probands had onset before age 20, 1 was in his twenties, and 16 were over 30 years old. It was hypothesized that the affected individuals with early-onset parkinsonism are homozygous or compound heterozygous, and the parents and other relatives with late-onset parkinsonism are heterozygous. For *parkin *analysis, we began with the family member from the generation with the earlier onset, so as to enrich for *parkin *mutations. We expected the majority of the index cases to be compound heterozygous. At the minimum, 16–49% of index cases should have had mutations, since this is the range reported for early-onset sporadic and familial parkinsonism [7,9,19]. However, only one case had *parkin *mutations. This patient had compound deletions in exon 3 with onset at age 8. Her sister, onset at age 15, also had the compound mutation. The relative with late-onset was an uncle with onset age of 64. Contrary to our hypothesis, the uncle did not have a *parkin *mutation. The cause of late-onset parkinsonism in the uncle was different from the nieces. In effect, we did not find any cases where we could attribute the intergenerational difference in age at onset to *parkin *dosage.

We also analyzed *parkin *in 28 additional patients with early-onset parkinsonism (onset age 14 to 40 years, mean 32.8 ± 7.0), either with (n = 4) or without (n = 24) family history, but with no evidence of anticipation. In this group, 3 probands had onset at or before age 20, 5 were in their twenties, and 20 were over 30 years old. Nine mutations were found in six individuals. Two subjects were compound heterozygous (onset ages 31 and 37) and four were heterozygous (onset ages 14, 25, 37, 37). The frequency of *parkin *carriers in early-onset parkinsonism without anticipation was 21% which is in the range 16% – 49% reported in the literature (the lower range represents population and clinic based studies similar to ours, while the higher frequencies were found in highly selected autosomal recessive families). It was of interest to determine if any of the parents of the three early-onset parkinsonism patients with compound mutations had developed late-onset parkinsonism. All six parents were heterozygous (5 were confirmed by genotyping, one was inferred), and all had remained free of parkinsonism to the ages of 53, 60, 63, 74, 76 and 78 yrs. These individuals may still develop parkinsonism.

## Discussion

Familial parkinsonism with anticipation may be more common than classical dominant and recessive subtypes combined. In our clinic population, among 487 patients studied, 145 had a first or second degree relative with PD; that is 30% which is in line with the published figures for other referral clinics. Among the 145 familial cases, 110 had parkinsonism in consecutive generations, which is compatible with autosomal dominant inheritance, and 35 had an affected sibling or cousin, which is suggestive of recessive inheritance. However, among the 110, only 26 were compatible with a classical dominant pattern (i.e., <10 years intergenerational variation in onset); while 63 exhibited 10–68 years of anticipation, and 7 had 10–17 years of reverse anticipation (14/110 had unknown onset ages). Our clinic is a referral center, which explains the relatively high proportions of early-onset and familial cases. The interesting finding was the relative proportions of autosomal dominant, autosomal recessive and anticipation cases within the familial subtype. Despite its relatively high prevalence, at least in our clinic, familial parkinsonism with anticipation has been largely overlooked in genetic research. A few parkinsonism families have been attributed to expansions in known SCA loci, but in the majority of the kindreds, the cause remains unknown. The more obvious possibilities are mitochondrial inheritance, modifier genes, parent-child exposure to environmental triggers, as yet unidentified triplet repeats or dominant genes with dosage effect, and in some cases, artifactual appearance of anticipation due to ascertainment bias. None of these are mutually exclusive; more than one may be true and operative in different families.

The current *parkin *literature suggest that possessing two *parkin *mutations is fully penetrant and leads to early-onset parkinsonism, whereas having only one mutation may be incompletely penetrant and lead to later disease onset. While the causative link to early-onset parkinsonism is widely accepted, the association of *parkin *with late-onset parkinsonism remains controversial [9,10,20,21]. We postulated that, if *parkin *heterozygotes are at risk for late-onset disease, then some *parkin *families should exhibit intergenerational variation in age at onset resembling anticipation, where heterozygous parents develop late-onset parkinsonism and children who inherit two mutations develop early-onset parkinsonism. We hoped to explain the appearance of anticipation in relation to *parkin *dosage, but the findings do not support this postulate. While the results rule out a link between *parkin *and anticipation in these families, they do not negate the association of *parkin *with late-onset parkinsonism.

## Conclusion

The phenomenon of anticipation is not due to *parkin *mutation dosage. The underlying mechanism for anticipation may be genetic or environmental. Identification and a-priori classification of pedigrees that exhibit significant intergenerational age at onset variation, as being distinct from families that display classical dominant pattern, may facilitate gene mapping studies by reducing heterogeneity. Anticipation in parkinsonism merits investigation in its own right, not only because it is a common phenomenon and may account for a large subset of familial parkinsonism, but it may also uncover a novel mechanism in parkinsonism.

## Competing interests

The author(s) declare that they have no competing interests.

## Authors' contributions

PP carried out the molecular genetic studies. LM interviewed the subjects and gathered family histories and medical records. JN performed the neurological examinations. GS participated in study design and supervised molecular genetic studies. JM performed molecular analysis of one large pedigree. HP conceived the study, participated in its design and coordination and drafted the manuscript. All authors read and approved the manuscript.

## Pre-publication history

The pre-publication history for this paper can be accessed here:


